# Eyes wide shut: Transcranial alternating current stimulation drives alpha rhythm in a state dependent manner

**DOI:** 10.1038/srep27138

**Published:** 2016-06-02

**Authors:** Philipp Ruhnau, Toralf Neuling, Marco Fuscá, Christoph S. Herrmann, Gianpaolo Demarchi, Nathan Weisz

**Affiliations:** 1 Center for Mind/Brain Sciences, University of Trento, via delle Regole 101, 38123 Mattarello, Italy; 2 Centre for Cognitive Neuroscience, University of Salzburg, Hellbrunnerstraße 34, 5020 Salzburg, Austria; 3 Experimental Psychology Lab, Center for Excellence “Hearing4all,” European Medical School, University of Oldenburg, 26111 Oldenburg, Germany; 4 Research Center Neurosensory Science, University of Oldenburg, Carl-von-Ossietzky-Strasse 9-11, 26129 Oldenburg, Germany

## Abstract

Transcranial alternating current stimulation (tACS) is used to modulate brain oscillations to measure changes in cognitive function. It is only since recently that brain activity in human subjects during tACS can be investigated. The present study aims to investigate the phase relationship between the external tACS signal and concurrent brain activity. Subjects were stimulated with tACS at individual alpha frequency during eyes open and eyes closed resting states. Electrodes were placed at Cz and Oz, which should affect parieto-occipital areas most strongly. Source space magnetoencephalography (MEG) data were used to estimate phase coherence between tACS and brain activity. Phase coherence was significantly increased in areas in the occipital pole in eyes open resting state only. The lag between tACS and brain responses showed considerable inter-individual variability. In conclusion, tACS at individual alpha frequency entrains brain activity in visual cortices. Interestingly, this effect is state dependent and is clearly observed with eyes open but only to a lesser extent with eyes closed.

Transcranial alternating current stimulation (tACS) is a revived and strongly used tool to investigate the causal role of brain oscillations on cognition and behavior. Many recent reviews have elucidated the importance of this tool in cognitive neuroscience^1^,^2^,^3^.

A substantial amount of evidence points to a direct influence of tACS on brain oscillations, mostly in the form of behavioral effects showing modulations of, for instance, detection ability of weak stimuli^4^, content in working memory^5^, memory task performance^6^, or crossmodal illusions^7^. All these studies applied tACS frequencies that had been linked to the investigated cognitive processes. Thus, it has been argued that tACS can show a causal relationship to cognitive function. However, this argument relies on the assumption that input waves and brain waves will align during tACS. Considering this strong assumption it is noteworthy that no proof in humans exists so far (but see^8^ for *in vitro* animal data).

Possibilities of investigating brain oscillations during tACS stimulation have been provided recently^9^,^10^,^11^. In a previous study^10^ we focused on the proof of principle aspect of recovering a well established alpha modulation (power increase when the eyes are closed vs. open) during tACS. Here, we use the same source space data to investigate the phase relationship of tACS signal and brain oscillations during different brain states. We stimulate subjects at their individual alpha frequency (IAF, ~10 Hz). Frequencies in the alpha range have been used as stimulation frequencies in many previous tACS studies^4^,^7^,^12^,^13^ because their dynamics are easily observable in most subjects in parieto-occipital areas while at rest.

In this work, we aim to investigate five major assumptions about the effects of tACS: 1) It is assumed that tACS leads to phasic entrainment, i.e. an alignment of brain activity and tACS phase^14^,^15^ and there is evidence for entrainment of neuronal activity from animal research^8^,^16^. Here, we estimate phase coherence of tACS signal and brain signal to investigate whether and how brain activity entrains online to tACS. 2) Strongest entrainment is suggested to happen in areas showing a ‘preference’ for the entraining frequency^15^,^17^. Thus, alpha frequency stimulation should be largest in visual cortex areas, the generators for visual cortical alpha. 3) Brain state dependent efficiency of tACS has been demonstrated for after-effects^14^, such that alpha-tACS affected endogenous alpha oscillations only when the subjects’ alpha activity was weak (eyes open), but not so when alpha activity was strong (eyes closed). A proof for state dependent effects *during* tACS is still missing, thus, we investigated data from eyes open and eyes closed resting states to follow up on that question. 4) Considerable between-subject variation has been found in behavioral effects of tACS^4^,^7^,^18^. To uncover the true tACS effects, phases have to be aligned across subjects to reveal group effects. Considering that electrical current travels through tissue instantaneously, this is surprising; however, it is likely a result of anatomical differences. We test whether entrainment of neural oscillations in visual areas during stimulation occurs with a phase offset (tACS to brain activity) in our data. This could provide a basis to explain between-subject differences in optimal phase.

## Material and Methods

### Subjects

Seventeen subjects (age M 28, SD 4; 8 female) were analyzed for the present study. All subjects gave written informed consent prior to the experiment. The study protocol was approved by the local ethics committee of the University of Trento, and the study was carried out in accordance with the approved guidelines.

### Procedure

Subjects sat in a comfortable chair in a dimly lit booth (AK3b, Vacuumschmelze, Germany) and were asked to fixate on a centrally presented cross without any further task (i.e. resting state) for two minutes with their eyes open and two minutes with their eyes closed. Resting state data were acquired in a sham condition and two individual alpha frequency (IAF) tACS blocks at different intensities (see below). The IAF was estimated from an eyes open resting state block, recorded before the start of the actual experiment, by identifying the peak frequency in the alpha range over occipital sensors (see Table S1 for details). The strong tACS condition was always after the sham and weak tACS blocks (counterbalanced) to avoid aftereffects.

### TACS parameters

We used a battery-operated stimulator system (DC-Stimulator Plus, NeuroConn GmbH, Ilmenau, Germany), which was placed outside the magnetically shielded room. A magnetic resonance imaging (MRI) module (NeuroConn GmbH, Ilmenau, Germany) connected the stimulator system and two conductive rubber stimulation electrodes (7 by 5 cm) centered at Cz and Oz, applied with a conductive paste (Ten20, D.O. Weaver, Aurora, CO, USA). The positions were chosen to maximally affect the parieto-occipital cortex^19^. Impedance values were kept below 15 kΩ (M 6.1 SD 0.8 kΩ). The stimulator delivered a sinusoidal current, without a DC offset, at the IAF. To keep subjects naive about the tACS stimulation, we stimulated below each subject’s sensation/phosphene threshold^10^. Average stimulation intensity was 0.65 mA (SD 0.45). In the weak tACS condition we stimulated at 0.05 mA, a stimulation intensity that should not affect neural activity^20^.

### MEG recording and analysis

Magnetoencephalography (MEG) was recorded from 306 sensors (102 magnetometers, 204 planar gradiometers) from 102 positions above the participants’ heads (Neuromag Vectorview, Elekta Oy, Helsinki, Finnland). The continuous data in each condition was band-pass filtered (1–200 Hz, 4^th^ order Butterworth), down-sampled to 512 Hz and then cut into 2 s long, non-overlapping epochs. For the tACS conditions, the epochs were aligned to a trough of the ongoing tACS under the occipital electrode.

To overcome the sensor level tACS artifact, the data were projected into source space by means of linearly constrained minimum variance (LCMV) beamforming^21^, applied to 889 equally-spaced (1.5 cm) points covering the whole brain, resulting in 889 virtual sensors. By using single-shell headmodels^22^ together with the lead field matrix and the covariance matrix (obtained from individual trials and averaged), we estimated beamformer filters which we subsequently multiplied with the epochs to obtain source level epochs. The used grid was in Montreal Neurological Institute (MNI) space and warped to the individual head space.

All analysis was accomplished with the open-source toolbox FieldTrip^23^ embedded in Matlab (Mathworks, Natick, USA).

### Phase coherence

First, epochs were averaged separately for the resting state (eyes open vs. closed) and tACS conditions (strong tACS, weak tACS, sham) for each subject. Then we obtained complex wavelet coefficients at the stimulation frequency via continuous Morlet wavelet transform between 0.5 to 1.5 s (in steps of 0.005 s) relative to epoch onset. Wavelets had a fixed width of 7 cycles. Phase (in radians) was then calculated with the Matlab function *angle*.

We computed phase coherence^24^,^25^ between the tACS reference signal (captured at a magnetometer under the occipital electrode, MEG2121) and all virtual sensors (889 grid points) for the tACS conditions, and between an artificial cosine (at tACS frequency) and all virtual sensors for the sham condition. Phase coherence (PC) is calculated as follows:


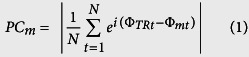


where N is the number of time points, Φ_*TRt*_ the phase of the tACS reference signal and Φ_*mt*_ the phase at virtual sensor *m* at time point *t*. Thus PC is coherence of the phase differences between tACS artifact and any virtual sensor over time. The same measure has recently been shown to be able to capture entrainment by tACS in the motor cortex^11^. To estimate the temporal lag between tACS and virtual sensors, we transformed the mean phase angle differences (tACS reference signal to virtual sensors) to millisecond values by multiplying them with 1000 and dividing them by 2*π* * *f*, where f is the IAF.

### Statistical analysis

To test whether there was increased PC during tACS compared to sham we compared values across the whole brain with Student’s t-tests (weak tACS vs. sham; strong tACS vs. sham) in the eyes open and eyes closed conditions. We used a permutation approach using cluster statistics that controls for the multiple comparison problem^26^. We ran 5000 randomizations, used the maximum sum per cluster as test statistic and set the alpha level to 5%.

To compare PC across all conditions in the visual cortex (mean over voxels along the Calcarine sulcus as selected using the Automated Anatomical Labeling [AAL] atlas) we computed a repeated measures analysis of variance (ANOVA) comprised of the factors resting state (eyes open; eyes closed) and tACS condition (sham, weak, strong). The Greenhouse-Geisser correction was applied to account for sphericity violations^27^. We report the corrected p-values and epsilon coefficient where appropriate. Post-hoc test were corrected using the false-discovery-rate (FDR) procedure^28^.

## Results

**Figure 1:**
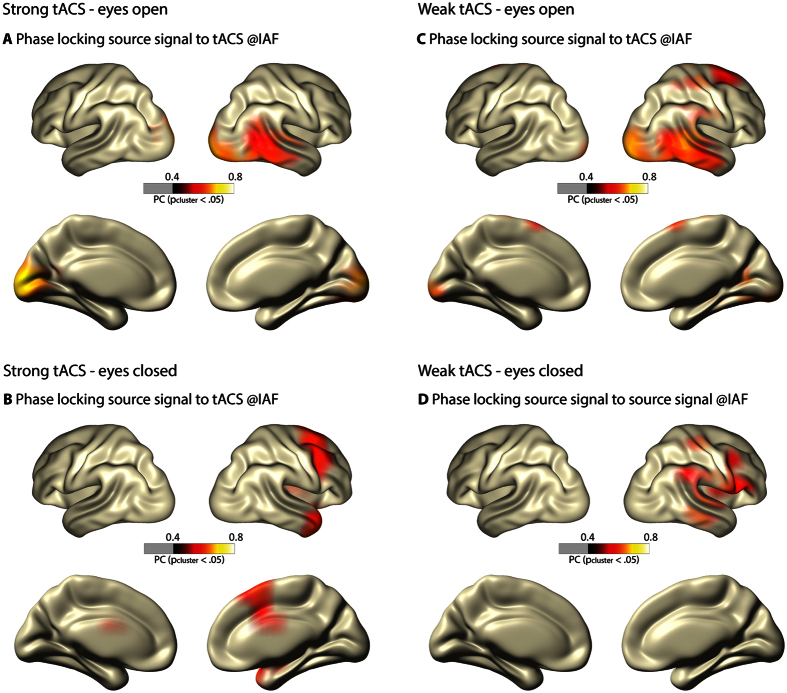
Phase coherence (PC) in the eyes open and eyes closed resting state conditions for weak and strong tACS. (**A,B**) show PC during strong tACS, (**C**,**D**) show PC during weak tACS. (**A**,**C**) show the eyes open state and (**B,D**) the eyes closed state. Brain activity shows increased PC in visual cortex areas with eyes open but not with eyes closed. All cortex maps are thresholded for significant differences (cluster corrected) in PC between tACS conditions and sham. An MNI template brain was used for visualization purposes.

With eyes open, weak and strong tACS resulted in increased PC compared to sham in the occipital pole including some right inferior temporal areas (both p_cluster_ < 2*10^−4^, Fig. 1A,C). Coherence values were largest in visual areas.

With eyes closed in the strong tACS condition, only superior frontal regions (p_cluster_ = 0.003, Fig. 1B) showed significant PC increases. With eyes closed in the weak tACS condition, regions covering right inferior frontal, pre- and post central areas as well as superior temporal cortex showed significant PC increases (p_cluster_ = 0.001, Fig. 1D).

**Figure 2:**
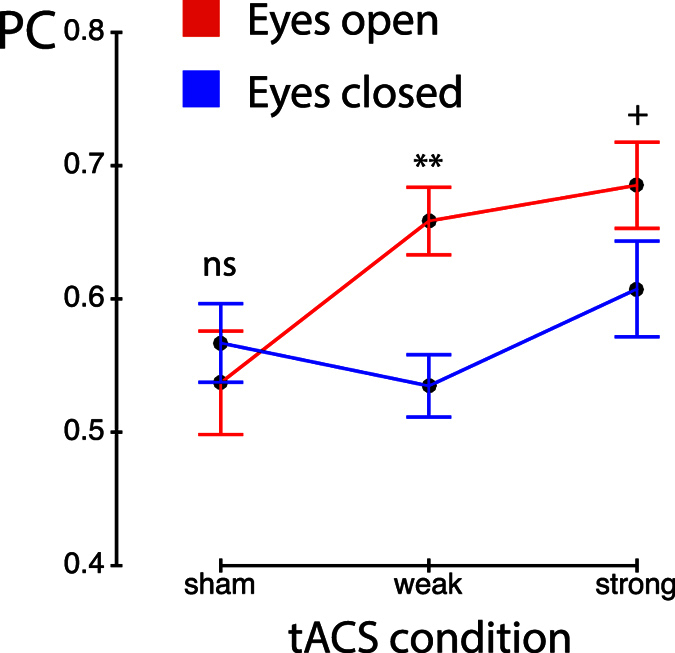
Phase coherence (PC) in visual cortex. Voxels in the Calcarine cortex were selected using the Automated Anatomical Labeling (AAL) atlas. PC increases with eyes open from sham to both tACS conditions (p_FDR_ < 0.01) but no tACS condition difference was found with eyes closed (p_FDR_ > 0.05). Error bars represent the standard error of the mean. ns – not significant, + - p_FDR_ < 0.1, **p_FDR_ < 0.01.

The ANOVA on PC amplitude in Calcarine areas (Fig. 2) showed a main effect of resting state (F(1, 16) = 4.77, p = 0.044) and a main effect of tACS condition (F(2, 32) = 4.50, p_GG_ = 0.021, ε = 0.952). These main effects were further explained by an interaction (F(2, 32) = 3.56, p_GG_ = 0.042,, ε = 0.961). Post hoc tests showed no differences in PC between the eyes open and eyes closed state in the sham condition (F(1, 16) = 0.47, p_FDR_ > 0.050) while PC was larger during eyes open compared to eyes closed in the weak tACS condition (F(1, 16) = 14.28, p_FDR_ < 0.010). The strong tACS condition showed a trend for the same effect (eyes open > eyes closed; F(1, 16) = 4.21, p_FDR_ < 0.100). Furthermore, the weak and strong tACS conditions yielded larger PC than sham in the eyes open state (F(1, 16) > 6.53, p_FDR_ < 0.05), however, no differences between tACS conditions (sham; weak; strong) were observed in the eyes closed resting state (all F(1, 16) < 3.09, p_FDR_ > 0.05).

**Figure 3:**
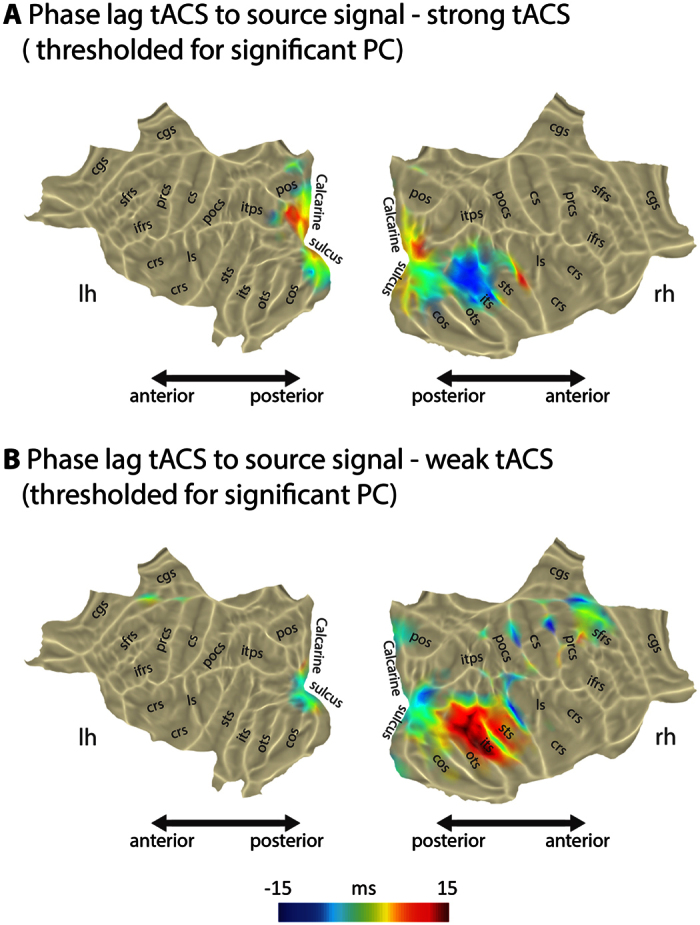
Phase lag of brain activity to tACS signal in the entrained regions in the occipital pole. The phase lag shifts along the visual cortex; there seem to be no instantaneous effects in the areas that are strongly phase locked. Individual subjects’ phase lag shows a substantial variation. The abbreviations for cortical sulci are based on the Mindboggle-101 dataset^31^.

We calculated the temporal lag in both weak and strong tACS conditions for the eyes open states, the conditions that showed significant PC in the visual cortex (Fig. 3). With strong tACS, the temporal lag in occipital areas as measured from the virtual sensor with highest PC was on average 6.0 ms, however, the variation across subjects was quite considerable (SD 28 ms, see also supplementary Figure S1). With weak tACS, the average temporal lag was −8.8 ms with again considerable variation (SD 25 ms). In both tACS conditions the temporal lag was different along visual cortex areas and not one constant value.

## Discussion

The present study proves that phase locking of neural oscillations to external alpha frequency tACS emerges in the visual system. Thus our first aim - to show entrainment of brain activity to tACS - was successful.

Our second goal was to test entrainment in regions showing a ‘preference’ for the entraining frequency^15^,^17^. Phase locking was strongest in visual cortex areas, where endogenous alpha activity can be localized. Considering that the current flow should affect broad regions along occipital and parietal areas^19^, this regional specific entrainment is evidence for entrainment in a region preferring the alpha rhythm.

Our third goal was to show whether brain state dependent effects that have been shown for after-effects of tACS^14^,^29^ can be found online. Indeed, we see robust tACS entrainment compared to sham in visual cortex areas when subjects had their eyes open (interestingly even with weak tACS stimulation, but see below), yet, no such increase was observed in visual cortex in eyes closed resting state data. We estimated the IAF based on eyes open resting state data at the beginning of the experiment^10^, thus, it seems natural that tACS at that frequency affects brain activity during a similar brain state. In reverse, this also means that alpha with eyes open represents a different state than eyes closed alpha, the latter less prone to perturbation by tACS. This finding might also provide an explanation under which circumstances tACS does not show effects. In a recent study^30^ no after-effects following multiple short tACS intervals (1 s) were found. The authors argue that these short intervals do not allow for neural plasticity to emerge, underlying tACS after-effects (see also^15^). Alternatively, they could be observing a state dependency effect. Namely they estimate the IAF in an eyes closed resting state block, however, tACS is applied while the subject is performing a visual task, thus the subject is in a different state, possibly not as susceptible to tACS. Likely, this interpretation is overly simplistic, yet, it allows for creating hypotheses that need to be tested in future experiments.

Our fourth goal, investigating individual phase entrainment differences, was based on previous behavioral findings on between subject variance when investigating neural phase during tACS^4^,^7^,^18^. Thus, we investigated the temporal lag between tACS and brain activity in visual cortex regions in those conditions that showed significant phase coherence in visual cortex (Fig. 3 and Figure S1). The entrained areas show an average temporal lag from tACS to brain oscillations of around 6 ms with strong and −9 ms with weak tACS; however, in both cases with large variation (Figure S1). This challenges the rather naive view that the tACS input wave can be directly translated into brain responses. Unfortunately, in the current experiment we did not record behavioral data. In future experiments, it is crucial to investigate behavioral and neural response together to see how well the temporal lag can predict the behavioral response within and across subjects. Here, we used individually adjusted tACS intensities, which could additionally explain temporal lag variation (even though in our study intensity did not correlate with temporal lag across subjects, see Table S1). However, further investigations are needed to explore how and if intensities can influence the tACS-brain activity lag.

Surprisingly, in data from a weak intensity tACS block (0.05 mA) also recorded during eyes open and eyes closed resting states, we found very similar patterns as with strong tACS. Specifically, we found increased entrainment in visual regions with eyes open but not with eyes closed. Such effects were not expected, considering that these intensities should not affect neural activity^20^ - the condition was planned as control condition. However, our results clearly raise the question of how low tACS intensities can be and still have an influence on brain activity. Modelling studies with higher intensities (1 mA) suggest that low intensity transcranial electrical stimulation reaches cortical areas because the effects scale linearly^19^. A preliminary correlation analysis on the tACS intensities across subjects (Figure S2) shows a trend to an increase in phase coherence with increasing intensity. However, our study was not designed for this question and more modelling studies are needed as well as a thorough follow-up that varies tACS intensities within subjects to investigate this further.

In conclusion, our results provide evidence for topographically specific entrainment of brain activity to alpha frequency tACS. In particular, entrainment was observed in a state dependent manner, mainly during eyes open resting state. Interestingly, entrainment occurs with subject specific phase lags between tACS and brain activity and also different lags within the visual system. Finally, our results challenge existing ideas that low intensity tACS will not affect neural activity.

## Additional Information

**How to cite this article**: Ruhnau, P. *et al*. Eyes wide shut: Transcranial alternating current stimulation drives alpha rhythm in a state dependent manner. *Sci. Rep.* **6**, 27138; doi: 10.1038/srep27138 (2016).

## Supplementary Material

Supplementary Information
