# Supplementary material for: Eyes wide shut: Transcranial alternating current stimulation drives alpha rhythm in a state dependent manner
Source: Sci Rep. 2016 Jun 2;6:27138. doi: 10.1038/srep27138 (PMC4890046; doi:10.1038/srep27138)
Supplement: Supplementary Information [file srep27138-s1.pdf]

Supplementary information

Eyes wide shut: Transcranial alternating current stimulation drives alpha rhythm in a state dependent manner

Philipp Ruhnau[1,2*], Toralf Neuling[1,2], Marco Fuscá[1], Christoph S. Herrmann[3,4], Gianpaolo Demarchi[1,2], Nathan Weisz[1,2]

1 - Center for Mind/Brain Sciences, University of Trento, via delle Regole 101, 38123 Mattarello, Italy

2 - Centre for Cognitive Neuroscience, University of Salzburg, Hellbrunnerstraße 34, 5020 Salzburg, Austria

3 - Experimental Psychology Lab, Center for Excellence "Hearing4all," European Medical School, University of Oldenburg, 26111 Oldenburg, Germany

4 - Research Center Neurosensory Science, University of Oldenburg, Carl-von-Ossietzky-Strasse 9-11, 26129 Oldenburg, Germany

* Corresponding author:
Philipp Ruhnau
Paris-Lodron University Salzburg
Division of Physiological Psychology
Hellbrunnerstraße 34
5020 Salzburg, Austria
Email: mail@philipp-ruhnau.de

Supplementary information

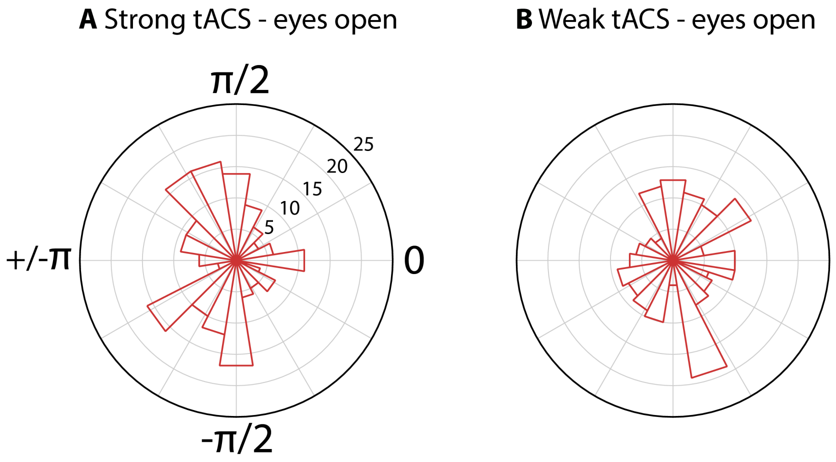

Figure S1 – Histograms of phase lag tACS to brain activity in the virtual sensors in visual cortex. Phase lag was estimated by computing the angle of the phase differences between tACS and brain activity. Considerable between-subject variation is evident with both tACS intensities.

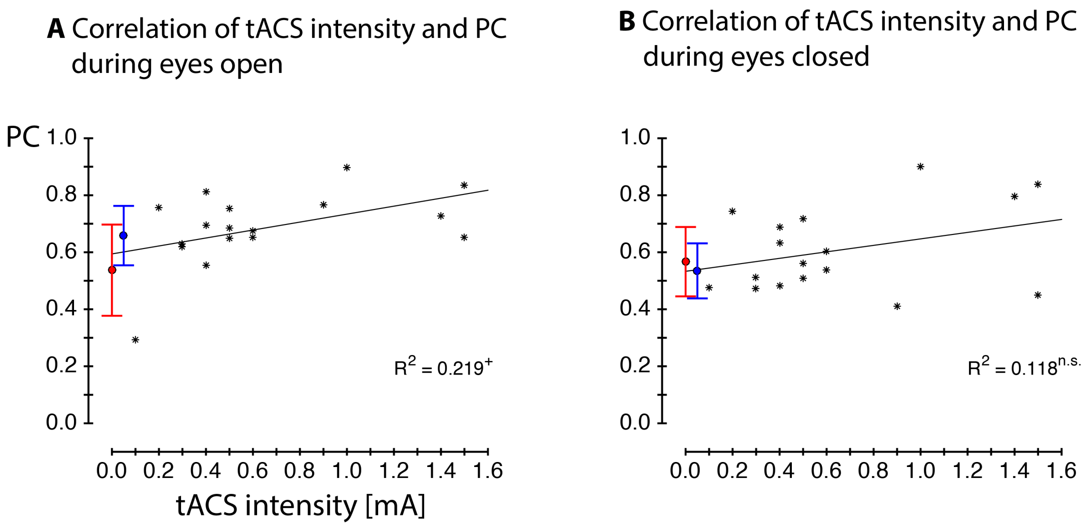

Figure S2 – Correlation of tACS intensity with PC in visual cortex in the strong tACS condition. A trend for a linear relationship is present in the eyes open resting state. Our data were not set to investigate this relationship – tACS intensity was not varied within subjects and many subjects cluster around a similar intensity of ~0.5 mA; however, these data are in line with previous data showing increasing tACS after-effects with increasing intensity and can be taken as a first indicator of the neuronal entrainment effects during tACS. The error bar at zero (red) represents mean PC and standard deviation in the sham condition. The error bar at 0.05 mA (blue) represents mean and standard deviation in the weak tACS condition.

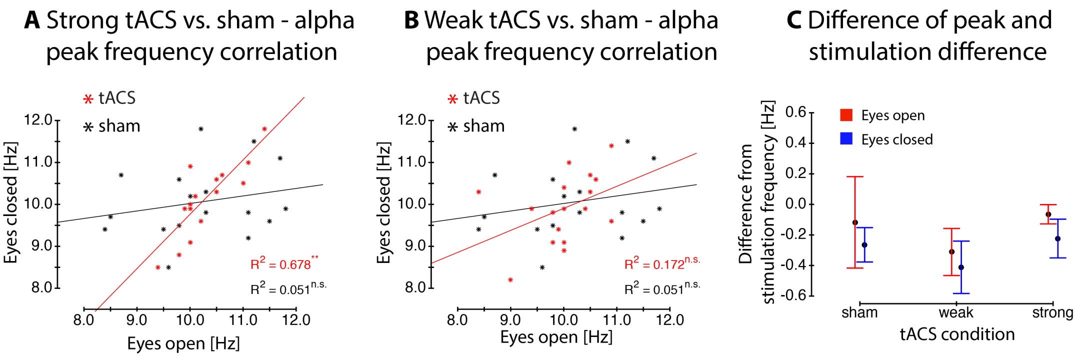

Figure S3 – Correlation of eyes open and eyes closed individual alpha frequency (here defined as maximum power value in the spectrum between 8 and 12 Hz in visual cortex virtual sensors). Panel A shows the correlation for sham and strong tACS and panel B for sham and weak tACS. During sham and weak tACS the correlation is not significant, probably also because of large variation during the eyes open resting state, which makes a reliable estimate difficult. As a result from entrainment the linear relationship is highly significant with strong tACS. Panel C shows the difference of peak frequency in all conditions from tACS stimulation frequency. Again, the eyes open sham condition shows strongest variation, which is highly reduced in the strong tACS condition.

Table S1 – Individual stimulation parameter and temporal lag.

IAF = individual alpha frequency, int = stimulation intensity, Z = impedance, tLag = temporal lag in the eyes open condition.

Correlations of tACS intensity with tLag:

weak tACS condition – $r = 0.19$, $p = 0.45$;

strong tACS condition – $r = 0.043$, $p = 0.87$

Subject	IAF (Hz)	intensity (mA)	Z(kΩ)	tLag (ms) – strong tACS	tLag (ms) – weak tACS
01	10	1.0	4.3	32	-43
02	9	0.6	2.8	12	34
03	10	0.4	4.3	34	43
04	10	0.5	9.0	-44	-18
05	10	0.9	14	-21	-28
06	10.5	0.6	9.8	15	-27
07	10.5	0.5	2.4	-31	-34
08	10	0.4	4.0	19	1
09	10	1.5	5.9	33	16
10	11	0.5	4.9	40	-14
11	11.5	0.1	7.0	-27	-8
12	10	0.4	9.9	7	-41
13	11.5	1.5	6.7	35	-7
14	10.5	0.2	4.2	-14	-18
15	10	0.3	3.9	-25	19
16	10	0.3	6.1	12	-3
17	10	1.4	2.7	28	-21
